# Supplementary material for: Emetine in Combination with Chloroquine Induces Oncolytic Potential of HIV-1-Based Lentiviral Particles
Source: Cells. 2022 Sep 10;11(18):2829. doi: 10.3390/cells11182829 (PMC9497060; doi:10.3390/cells11182829)
Supplement: Supplementary file 1 [file cells-11-02829-s001.zip › cells-1825596-supplementary.pdf]

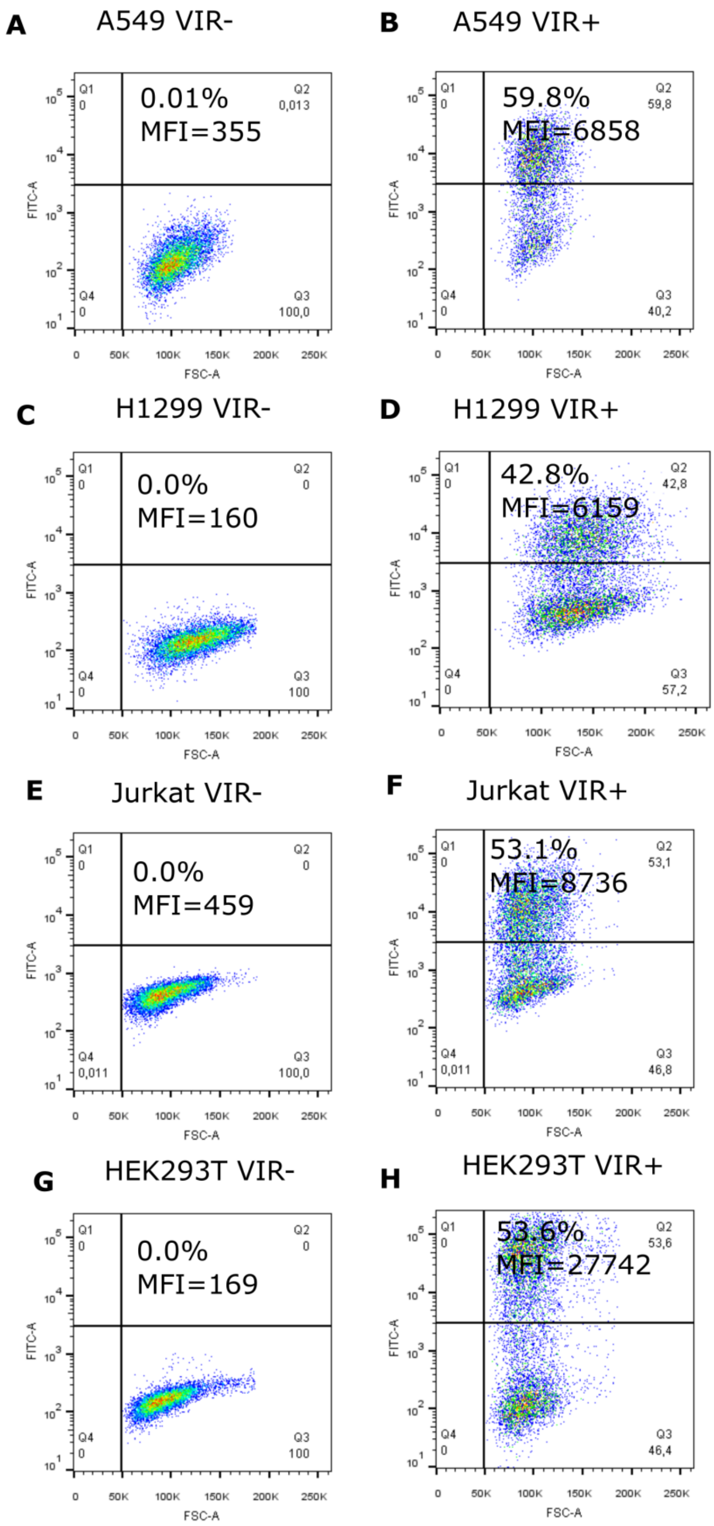

Supplementary Figure S1. The percentage and MFI of cells transduced with GFP encoding virus. The dot blot panels on the left part of the Figure represent the absence of GFP positive cells when no virus were added (VIR-); The panels on the right part of the figure represent the absolute percentage of GFP positive cells transduced with virus encoding GFP not treated with drugs used for normalisation of the samples treated with drugs. For every cell line (A549, H1299, Jurkat and HEJ293T) these MFIs and percentage obtained from the analysis of three replicates were taken as 100%.

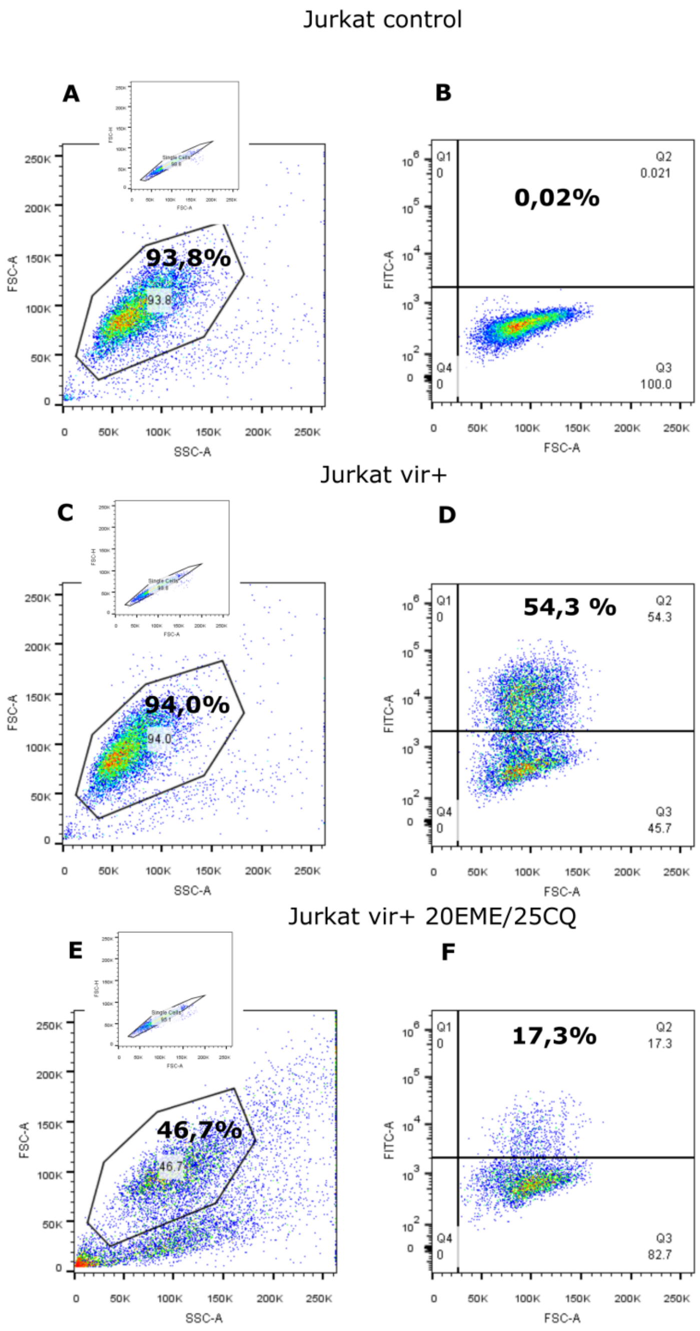

Supplementary Figure S2. An example of gating of GFP positive Jurkat cells transduced with replication deficient lentiviral particles. **A.** The percentage of cells with «normal» FSC and SSC representing the population of native Jurkat cells. Upper small dot blot represent the population of single cells used to detect the GFP positive cells. **B.** The percentage of GFP positive cells in single cell population were analysed in native Jurkat cells not transducted by replication deficient lentiviral vectors RD-HIV1 (Jurkat control). **C.** The gating of cells transduced with GFP encoding RD-HIV1 particles (Jurkat vir+). **D.** Absolute percentage of GFP positive cells in population. This percentage was used for normalization and represented on bar blots (Figure 2 of the manuscript) as 100%. **E.** The population of cells treated with 20nM EME and 25uM CQ and infected with RD-HIV1, representing that the percentage of cells with «normal» FSC and SSC comparative with native cells becomes twice lower. The amount of events with lower FSC, what is similar to dead cells became more representative. **F.** The percentage of GFP positive cells with «normal» FCS and SSC pre-treated with combination of EME and CQ (Jurkat vir+ 20EME/25CQ). This percentage was normalized to the percentage of GFP positive cells not pre-treated with CQ and EME.

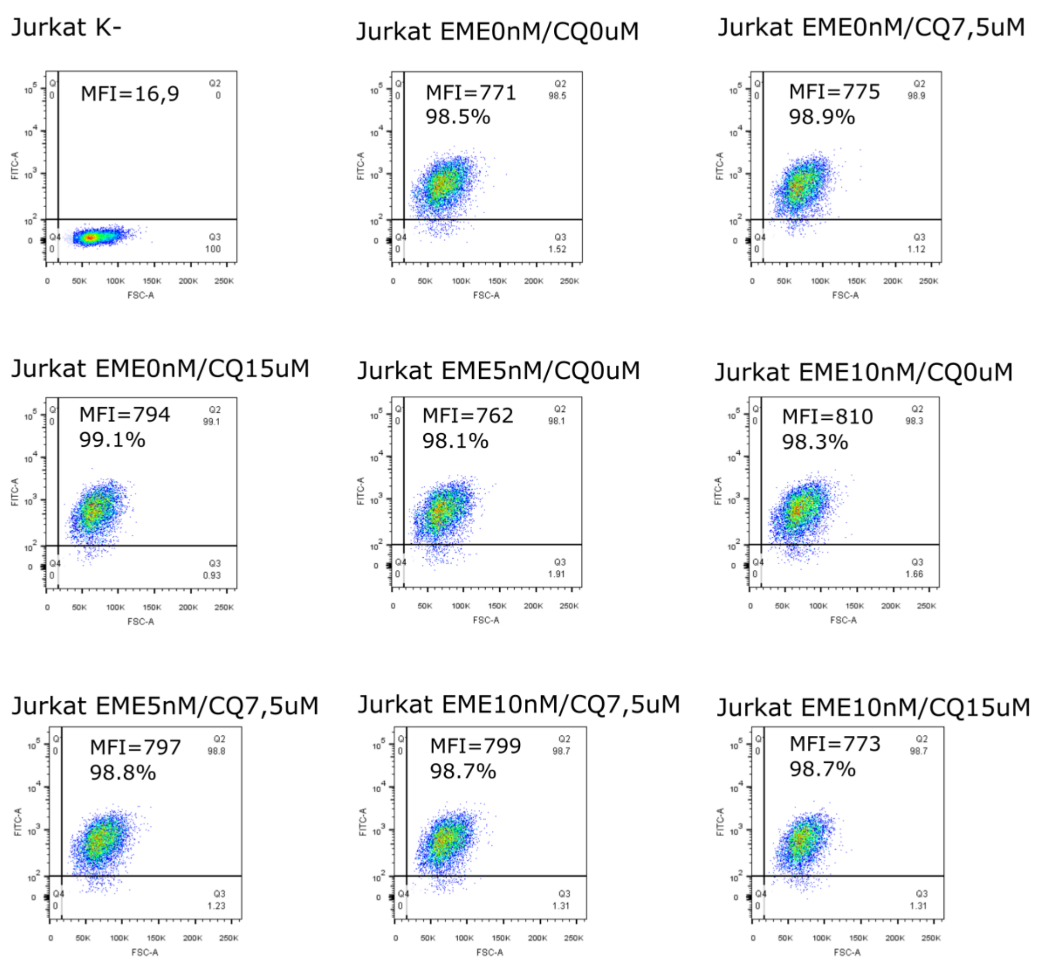

Supplementary Figure S3. Emetine and Chloroquine do not affect GFP in Jurkat cells which were stably transduced with replication deficient GFP encoding vector Lego G2 (the same, which was used in our study). Seven days after establishment of this model cell line we treated the cells with Emetine or Chloroquine alone and in combinations for 72 hours and measured the percentage of fluorescent cells and their MFI using FACS. No changes in fluorescence of cells were detected. This may indicate that Emetine (in nM concentrations) or Chloroquine (uM concentrations) taken alone or in combination do not affect GFP protein in cells or affect it synthesis.
The dot plots represent the transduction efficiency and MFI of cells treated with various concentrations of EME (Emetine) and CQ (Chloroquine) and their combinations. Jurkat K- is a negative control of cells not treated with virus and drugs.

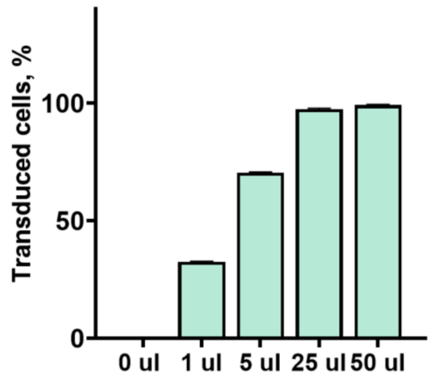

Supplementary figure S4. The percentage of transduced Jurkat cells plated in 96-well plates when GFP encoding replication deficient HIV-1 particles were added (1-50uL).

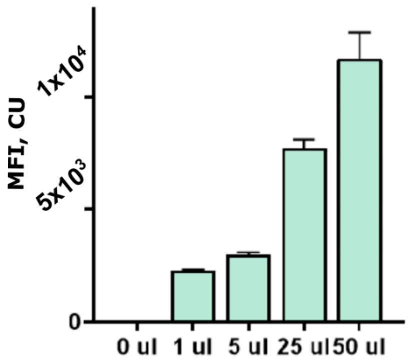

Supplementary figure S5. The MFI (mean fluorescence intensity) of transduced Jurkat cells plated in 96-well plates when GFP encoding replication deficient HIV-1 particles were added (1-50uL).

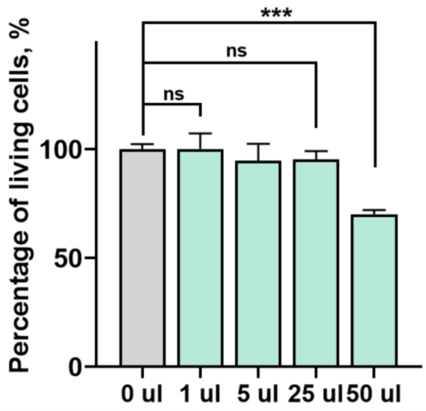

Supplementary Figure S6. The percentage of living Jurkat cells plated in 96-well plates when GFP encoding replication deficient HIV-1 particles were added (1-50uL). The grey bar represents the percentage of living cells not treated with virus. The amount of cells was counted three days post transduction.

Comments to Supplementary Figures S4-S6. The increase of the volume of virus containing stocks to achieve 80%-90% efficiency (Supplementary Figure S4) also not affect the survival of transduced cells (Supplementary Figure S6). Only the significant increase of the volume of the virus containing stock leading to increase in MFI (Supplementary Figure S5) at the stably high percentage of transduced cells (more than 95%), which indicate the increase in multiplication of infection, caused about 20% decrease in cell count (Supplementary Figure S6).
